# Stem Cells and Their Mediators – Next Generation Therapy for Bronchopulmonary Dysplasia

**DOI:** 10.3389/fmed.2015.00050

**Published:** 2015-07-30

**Authors:** Marius A. Möbius, Bernard Thébaud

**Affiliations:** ^1^Department of Neonatology and Pediatric Critical Care Medicine, Medical Faculty, University Hospital Carl Gustav Carus, Technische Universität Dresden, Dresden, Germany; ^2^DFG Research Center and Cluster of Excellence for Regenerative Therapies (CRTD), Technische Universität Dresden, Dresden, Germany; ^3^Regenerative Medicine Program, Sprott Centre for Stem Cell Research, Ottawa Hospital Research Institute, University of Ottawa, Ottawa, ON, Canada; ^4^Division of Neonatology, Department of Pediatrics, Children’s Hospital of Eastern Ontario, University of Ottawa, Ottawa, ON, Canada

**Keywords:** bronchopulmonary dysplasia, lung, stem cells, mesenchymal stromal cells, endothelial progenitor cells, current good manufacturing practice, potency assay

## Abstract

Bronchopulmonary dysplasia (BPD) remains a major complication of premature birth. Despite great achievements in perinatal medicine over the past decades, there is no treatment for BPD. Recent insights into the biology of stem/progenitor cells have ignited the hope of regenerating damaged organs. Animal experiments revealed promising lung protection/regeneration with stem/progenitor cells in experimental models of BPD and led to first clinical studies in infants. However, these therapies are still experimental and knowledge on the exact mechanisms of action of these cells is limited. Furthermore, heterogeneity of the therapeutic cell populations and missing potency assays currently limit our ability to predict a cell product’s efficacy. Here, we review the therapeutic potential of mesenchymal stromal, endothelial progenitor, and amniotic epithelial cells for BPD. Current knowledge on the mechanisms behind the beneficial effects of stem cells is briefly summarized. Finally, we discuss the obstacles constraining their transition from bench-to-bedside and present potential approaches to overcome them.

## Introduction

The proper ventilation and oxygenation of a premature newborn is the foremost task in neonatology. But from the first breath of a premature newborn in the delivery room to the spontaneous or mechanical ventilation on the Neonatal Intensive Care Unit, the immature lung is always exposed to a non-physiological substance; it is not prepared for at this age: air, containing at least five times the oxygen concentration of the amniotic fluid (1). The abrupt confrontation of the immature lung to this and other hostile extrauterine conditions leads to the chronic lung disease of prematurity or bronchopulmonary dysplasia (BPD).

Despite advances in the management of premature infants, respiratory complications still account for approximately one-quarter of all NICU deaths (2). BPD, characterized by impaired lung growth, remains the most common complication of premature birth (3, 4). Currently, there is no effective treatment for BPD and all present approaches remain either supportive, present major adverse effects (steriods) or show only small benefits (vitamin A, caffeine).

Cell-based therapies may open a completely new chapter in the therapy of BPD. Over the past years, animal studies using stem and progenitor cells as therapeutics showed very promising results, which have lead to first trials in human (5). This review summarizes our current knowledge about the therapeutic potential of these genuine facilitators of lung growth and regeneration.

## Stem Cells – Origin of Growth, Repair, and Disease

Stem or progenitor cells reside in virtually all tissues at all stages of development. They are generally defined by the ability to (**I**) undergo self-renewal and (**II**) give rise to more differentiated cells. The extent to which these cells can differentiate is called potency. Stem cells harbor the potential to differentiate into placental and embryonic tissue (*totipotent* stem cells of the morula stage) and along the various embryonic germ layers (*pluripotent*, embryonic stem cells). They further give raise to several adult cell types (*multipotent*, i.e., hematopoietic stem cells). Conversely, progenitor cells are thought to give raise to only one specific adult cell type (*unipotent*, i.e., type 2 alveolar epithelial cells).

Toti-, pluri-, and multipotent stem cells enable early development of the embryonal structures and subsequent organ differentiation until the beginning of the fetal period approximately 8 weeks *post conceptionem*. After this period, derivates of these cells can be found as resident stem or progenitor cells in virtually all fetal and adult tissues deriving from all three germ layers, including the bone marrow (6), gut (7), brain (8), and lung (9).

Their major task is the facilitation of growth and of tissue regeneration and maintenance, e.g., providing new, differentiated cells after cell loss due to normal usage or injury while remaining in a proliferative, lesser differentiated state on their own (self-renewal). This happens to various extends. Some tissues – such as the gut or bone marrow – contain stem cells with high proliferative and regenerative capacity, while others – such as the brain and the heart – grow until adulthood, but have only limited regenerative potential once damaged.

The lung is a complex organ deriving from endodermal and mesodermal origin and harbors several endodermal (epithelial) and mesodermal (mesenchymal and endothelial) stem and progenitor cell types (10), each of them with different capabilities to differentiate and proliferate. As of now, over 40 different lung cell types have been described; numerous of them exert more or less characteristics of stem cells (9–11).

Since enabling growth and regeneration is the main role of stem or progenitor cells in non-embryonic tissues, organ failure would suggest a pathology of the organ resident stem cell population(s). Indeed, several events before (prolonged rupture of the membranes, oligohydramnios, severe intrauterine growth restriction, congenital diaphragmatic hernia/CDH) or after birth (mechanical ventilation, oxygen) may impair stem cell function. Lung diseases with abnormal growth of lung compartments, such as the bronchiolitis obliterans syndrome (BOS) following lung transplantation (12) or lung hypoplasia following CDH (13), can be linked to dysfunction of the resident progenitor cells.

In BPD, qualitative or quantitative impairment of resident mesenchymal and endothelial stem or progenitor cells seems to contribute to the disease pathogenesis or to the incapacity of the lung to repair itself (11). Less is known about the pathogenic role of stem or progenitor cells in the endodermal, epithelial lung compartments, such as the bronchioalveolar stem cells (BASCs) (14).

Conversely, exogenous stem cells or their products derived from the mesenchymal (14–26), epithelial (27–29), or endothelial (30, 31) compartment of easily accessible tissue, such as the bone marrow, placenta, or the umbilical cord prevent or restore lung damage in animal models of BPD. Most of these data have been generated in neonatal rodents exposed to hyperoxia, a model which will be discussed below. Newer models combining several factors contributing to BPD [such as antenatal hypoxia, inflammation, and mechanical ventilation (32, 33)] will be useful to assess the pathophysiology of BPD and therapeutic benefit of cell therapies more completely. Various cell therapies have been proposed (34), and the following paragraphs will focus on the most extensively explored therapeutic stem cells for BPD: mesenchymal stromal cells (MSCs), endothelial progenitor cells (EPCs, including endothelial colony forming cells, ECFCs), as well as amnion epithelial cells (AECs).

## MSCs as Therapeutic Cells

Mesenchymal stem or stromal cells (MSCs) are the most promising cells in regenerative medicine. Their therapeutic potential is currently investigated in virtually every disease one can think of. As of February 2015, PubMed lists over 37,500 references for these cells; almost double the number from 2012 (35).

First described in hematopoietic tissues by Friedenstein and his colleagues in 1970 (36), MSCs have been identified in adult organs deriving from the mesodermal germ layer, including the bone marrow and adipose tissue. Furthermore, they can be found in fetal-restricted mesodermal derivates like the umbilical cord stroma and cord blood as well as in the placenta and the amniotic fluid [comprehensively reviewed by Hass and colleagues (37)]. Interestingly, MSCs have also been identified in tissues deriving from the (ectodermal) neural crest, such as the mandibula (38).

Cord-derived MSCs from the Wharton’s Jelly are of particular interest for the treatment of neonatal diseases. Indeed, the umbilical cord stroma is

- readily available at birth and thus clinically relevant
- with 100 million births worldwide a large source of stem cells
- safe and painless to the mother and her child as cells are harvested after delivery from otherwise discarded tissue and thus devoid of ethical dilemma
- importantly, these cells hold superior healing capabilities compared to adult bone marrow cells (39).

As implied by their multiple residence tissues, MSCs represent a very heterogeneous cell population (40, 41). MSCs from one source exert different properties than MSCs from another (37, 42). Some cells within the MSC population are true stem cells with the potential to undergo complete self-renewal and some are not. Therefore, the global population of MSCs should be identified as “mesenchymal stromal cells” rather than “mesenchymal stem cells” (35).

The minimal criteria to define a MSC (41) are widely accepted, but relatively loose and include the following four:

- The ability of the cell to adhere and grow on plain, uncoated, tissue culture treated plastic surfaces, e.g., the ability to secrete large amounts of extracellular matrix.
- The presence of CD73, CD90 (thymocyte antigen *thy-1*), and CD105 (*endoglin 1*) on the cell’s surface.
- The absence of the surface markers CD34, CD45, CD14/CD11b, CD19/CD79*α*, and HLA-DR, which label various cell lines from the hematopoetic lineage.
- The ability of the cells to differentiate along adipogenic, osteogenic, and chondrogenic lineages when stimulated *in vitro*.

These criteria were initially created to define MSCs derived from the bone marrow, where they need to be distinguished from the hematopoietic stem and progenitor cells giving rise to the blood cell lines. But as mentioned above, MSCs can also be found in other organs and tissues where they need to be distinguished from resident, mature fibroblasts, endothelial cells, and other non-hematopoetic cell types. Therefore, additional criteria for defining potentially therapeutic MSCs from, i.e., the umbilical cord or the adipose tissue, are required and currently under development. Several additional surface markers including CD10, CD29, CD106, CD146, CD166 or CD200 (42), and CD271 (43) have been proposed.

Bone marrow-derived MSCs exert a robust differentiation potential along osteogenic, chondrogenic, and adipogenic lineages. Conversely, some MSC populations can be differentiated into epithelial (44, 45), endothelial (46), and neural cells (47) while lacking the ability to differentiate along certain other, i.e., chondrogenic lineages (35). Therefore, criteria for a characterization by trilineage differentiation may need to be revised as well.

Functional tests, such as the assessment of the cell’s immune-regulatory properties (48) and their secretome (49) following specific stimuli, gain importance and will open a new avenue for a functional, rather than a morphological description of potentially therapeutic MSC products. Nevertheless, a single, striking marker or feature to define an MSC has not yet been found; neither is there a valid test to assess the “stemness” or “therapeutic potential” of such a cell, a major problem, which will be discussed below.

### Lung-resident MSCs and the development of BPD

Our current understanding of normal alveolar growth and the cellular and extracellular mechanisms behind its regulation suggest a crucial role of tissue-resident lung stem cells from mesenchymal, endothelial, and epithelial origin in this complex process (50, 51). Therefore, damage to the resident lung stem or progenitor cells – by inflammation, hyperoxia, malnutrition, shear stress, or other influences – may results in a loss or severe impairment of endogenous growth and regeneration potential.

The lung-resident MSC may play a critical role as regulator of lung development, coordinating epithelial and endothelial growth (52). When these cells become damaged in preterm infants, lung development gets out of sync leading to BPD. The properties of human neonatal and fetal lung MSCs are currently under investigation. While resident lung MSCs are by far not as well described as, i.e., BM-MSCs or adipose tissue-derived MSCs, pioneering work by Dr. Hershenson’s group found that the presence of MSCs in the tracheal aspirates of ventilated preterm infants predicted BPD (53–55).

These cells express less platelet-derived growth factor-receptor alpha (PDGFR-α) as compared to MSCs from babies without BPD (56). Furthermore, they present a profound autocrine production of transforming growth factor beta 1 (TGF-1) (57) and increased β-catenin signaling (58). The disruption of these pathways controlling the myofibroblastic differentiation (PDGFR-α, TGF-β1, and β-catenin) leads to disrupted formation of alveolar tips and interstitial lung fibrosis (58, 59). Moreover, the function of specific lung-resident stem cells with mesenchymal, endothelial, and epithelial differentiation potential (lung side population cells) (60) is disrupted in murine hyperoxia-induced lung injury (61).

Therefore, these findings suggest that damage to endogenous MSCs may contribute to the disease pathogenesis of BPD. Conversely, exogenous MSCs show consistent therapeutic benefits in experimental neonatal lung injury models. How these exogenous MSCs affect resident lung MSCs is unknown.

### Therapeutic benefits of exogenous MSCs

The beneficial effects of exogenous MSCs have best been described in hyperoxia-induced rodent models mimicking BPD (33, 62, 63). Rodents are convenient because they are born at the saccular stage of lung development, which corresponds to the lung developmental stage of a human infant born at 26–28 weeks of gestation (62). To summarize the models in brief, term born rodents are exposed to hyperoxia (FiO_2_ 0.60–0.95) for 1–2 weeks; rats or mice subsequently develop structural lung changes consistent with pathological findings of human infants that died with BPD (64). Alveolar simplification, capillary rarefaction, and leakage with extravascular fibrin and plasma protein accumulation, lung fibrosis with increased collagen and disordered elastin deposition, pulmonary hypertension, as well as influx of inflammatory cells can be observed (33, 62, 63).

A second model using prematurely delivered baboons model at 125 days and mechanically ventilated for 2 weeks offers unique opportunities to test promising (stem cell-based) therapies in a model close to the clinical setting (65). Due to the close relationship to man, long-term effects of treatment on growth and development can easily be observed, giving valuable information for clinical applications in premature human infants.

Mesenchymal stromal cells have striking beneficial effects in the hyperoxia-induced model of BPD. In 2007, Tian et al. (26) reported that intravenous injection of bone marrow-derived MSCs ameliorates the oxygen-induced neonatal lung injury. Two papers published simultaneously by Aslam et al. (24) and van Haaften et al. (25) in 2009 demonstrated that MSCs derived from the bone marrow of healthy, adult rodents prevent oxygen-induced neonatal lung injury.

Both authors administered MSCs on postnatal day 4 before exposing the pups to hyperoxia to assess the preventive potential of the cells. Aslam and colleagues administered 5 × 10^4^ cells (approximately 5 × 10^6^ cells/kg bodyweight) intravenously, whereas van Haaften et al. used an intratracheal administration route and applied double the dose (1 × 10^7^ MSCs/kg bodyweight). A significant decrease in alveolar wall thickness as well as an increase in vessel density and alveolar septation was observed in both studies. Furthermore, increased exercise capacity and reduced pulmonary hypertension was noted (18, 25).

Remarkably, very few of the injected cells were retained in the lung, indicating that cell engraftment contributes minimally – if at all – to the therapeutic benefit of MSCs. The intratracheal, intraperitoneal, or intravenous administration of cell free conditioned medium (CdM; concentrated tissue culture supernatant of MSCs) showed beneficial effects comparable to whole cell therapy. However, as no reliable methods to describe and normalize doses and composition of CdM have been utilized, a direct comparison of the two therapy regimens is inaccurate.

These experiments have been repeated several times with MSCs from the rat or human bone marrow (14, 17, 18, 21) or human cord blood (16, 22, 23, 66) and their respective conditioned media with similar results [reviewed by Fung et al. (67)]. Furthermore, recent pre-clinical studies by Chang and colleagues investigated the influences of the dose (22), timing (66), and administration route (23) of MSCs in a rat model of BPD. These studies favor an early, intratracheal administration of 0.5–5 × 10^7^ MSCs/kg bodyweight.

MSCs and their CdM were also able to rescue hyperoxia-induced lung injury (16). Moreover, the beneficial effects of a treatment with these cells are not transient. Adult rats that received MSCs in their neonatal period before (16), during (17), or after exposure (16) to hyperoxia show persistent improvements in lung architecture, exercise capacity, and vascularization in long-term follow-up studies up to 6 months.

The exact mechanism behind the effects remains unclear. Secreted anti-inflammatory proteins, angiokines, and other lung protective substances including stanniocalcin-1 (19, 68), prostaglandin E2 (12), and TNF-stimulated gene/protein 6 (TSG-6) (69–71) are strongly suggested to account for the short-term effects and protect the lungs against the acute injury. These substances secreted by the MSCs blunt the immediate and oblique injury effects like the influx of inflammatory cells and their associated deleterious effects. This has not only been described in neonatal hyperoxic models but also in several other experimental studies using bleomycin (72), lipopolysaccaride (73), ovalbumin (74), or prolonged ventilation (75) to challenge the lung.

The pathophysiology of BPD is not limited to inflammation, despite a major contribution of this process to the development of the disease (76). BPD is a multi-factorial disease and the characteristic and life-impairing feature of BPD – compromised alveolar growth beyond the neonatal period – can best be explained by a persistent impairment of the mechanisms regulating lung growth and development, including the resident stem/progenitor cells.

### The M&M’s of therapeutic cells – microvesicles and mitochondria in long-term effects of MSCs

As described above, very few cells engraft in the lung (25). The engrafted cells die rapidly and are not detectable with quantitative PCR methods or high-specific stainings after a few weeks when xenogeneic MSCs (=cells from a different species) were used (16). Authors using an allogeneic approach described a comparably low, but prolonged engraftment (up to 100 days) into the alveolar wall with potential transdifferentiation into surfactant-protein C producing cells (17, 25).

However, these events are very rare and do not contribute to the therapeutic effect of MSCs *in vivo* [reviewed by Kotton and Fine (77)]. Engraftment and transdifferentiation of MSCs may be considered as artifacts of the immunohistochemical detection method (78).

#### Microvesicles as Carriers of Therapeutic Agents

As discussed previously, secreted proteins mainly account for the short-term effects of transplanted MSCs or their CdM. But a long-term effect on the lung cells cannot be explained by just a single administration or secretion of cytokines. Extracellular vesicles, small microparticles containing nucleic acids, proteins, and lipids (79) may answer this question. Specific subtypes of these particles – so-called exosomes – are secreted by numerous cell types, including MSCs (80). They harbor the potential to reduce inflammation and blunt hypoxia-induced pulmonary hypertension (80) as well as to ameliorate endotoxin-induced lung injury (81).

Exosomes are, besides cytokines and other secreted proteins, the potential therapeutic components of conditioned medium. As reviewed comprehensively by Colombo et al. (79), exosomes can be taken up into the target cell by various mechanisms. Specific nucleic acids – so-called microRNA (82) – can transpose to the nucleus and silence specific genes for long periods (83) or interfere with the protein translation. These mechanisms could account for long-term beneficial effects on damaged lung cells in BPD.

#### Therapeutic Mitochondrial Transfer in Lung Disease

Another mechanism contributing to the long-term efficacy of MSCs may be the transfer of mitochondria from MSCs to damaged lung cells. Mitochondrial dysfunction plays a critical role in the development of experimental BPD in primates (84) and rodents (85–88).

In 2006, mitochondrial transfer from MSCs to other cells *in vitro* was described (89). Recent *in vivo* studies revealed that mitochondrial transfer plays a crucial role in animal models of lung injury. Intratracheally administered MSCs form microtubes and transpose mitochondria toward damaged alveolar type II cells, which leads to higher alveolar ATP-content and profound protection against lipopolysaccharide-induced acute lung injury (90). In chronic lung injury, therapeutic cells were able to reduce the alveolar damage as well as the interstitial fibrosis by mitochondrial transfer (91). Data supporting the role of mitochondrial transfer in neonatal chronic lung disease are pending.

### Safe, efficacious, effective? MSCs in clinical studies

These promising laboratory studies have lead to early phase clinical trials exploring the feasibility and safety of MSCs in various pulmonary diseases (Table 1). Chang et al. recently completed the first phase I dose escalation study using allogeneic human umbilical cord blood-derived MSCs in 9 preterm infants at risk of developing BPD (5). They administered 1 × 10^7^ or 2 × 10^7^ MSCs derived from the cord blood of healthy-term infants intratracheally and observed no serious adverse events or acute toxicity of the cells. Currently, several follow-up studies evaluating the long-term effects of the administered cells are listed on www.clinicaltrials.gov, and a placebo-controlled phase II trial (NCT01828957) is recruiting patients.

**Table 1:** **MSCs in clinical trails for pulmonary diseases**.

Condition	Phase	Design	Number of participants	Cell origin	NCT ID
Adult ARDS	I	Open	10	bm-msc (allo)	NCT02215811
	I	Randomized, double-blind	9	bm-msc (allo)	NCT01775774
	II	Randomized, placebo-controlled, double-blind	60	bm-msc (allo)	NCT02097641
	I	Randomized, placebo-controlled, double-blind	20	at-msc (allo)	NCT01902082
Air leakage after lung resection	I/II	Open	10	msc N/S (auto)	NCT02045745
Asthma	I/II	Open	20	cdm-uc (allo)	NCT02192736
BPD	I^‡^(5)	Open	9	ucb-msc (allo)	NCT01297205
	I	Open	12	ucb-msc (allo)	NCT02381366
	II	Randomized, placebo-controlled, double-blind	70	ucb-msc (allo)	NCT01828957
COPD	II^‡^(92)	Randomized, placebo-controlled, double-blind	62	bm-msc (allo)	NCT00683722
IPF	I	Open	18	bm-msc (auto)	NCT01919827
	I^‡^	Open	8	pla-msc (allo)	NCT01385644
	II	Randomized, open	60	at-msc (auto)	NCT02135380
BOS after lung transplantation	I	Open	9	bm-msc (allo)	NCT02181712
	I	Open	10	msc N/S (allo)	NCT01175655
Pulmonary emphysema	I/II	Randomized, open	30	bm-msc (allo)	NCT01849159

*Ongoing and completed interventional clinical trails listed on www.clinicaltrials.gov using mesenchymal stem or stromal cells to treat diseases of the lung*.

*^‡^Completed trials are marked with a diesis*.

*pla-msc, placenta-derived MSCs; *bm-msc*, bone marrow-derived MSCs; *ucb-msc*, umbilical cord blood-derived MSCs; *at-msc*, adipose tissue-derived MSCs; *cdm-uc*, conditioned media from umbilical cord-derived MSCs; *msc N/S*, source of cells not specified; *allo*, allogenic cells; *auto*, autologous cells*.

Clinical studies with MSCs are warranted. Obviously, MSC therapy in the neonatal population requires extremely careful risk-benefit considerations. Lessons learned from large, placebo-controlled phase III clinical trials using MSCs in steroid-refractory graft-versus-host disease (GvHD) (93) suggest that despite very promising results in animal models and phase I and II studies (94) current MSC preparations have no predictable therapeutic effect. Therapy with MSCs is complex and influenced by more factors than other cellular therapies, such as blood transfusions or hematopoietic stem cells for bone marrow transplantation.

#### MSCs – a Pharmaceutical Product in the Making

A major problem for clinical trials is the heterogeneity of the cell population termed MSCs. The markers and features defining an MSC are still evolving. As outlined previously, the cells characteristics, such as surface marker-, protein- and gene expression vary with the source, isolation, culture and expansion methods and donor age (35). Virtually every laboratory established (and patented) its own protocols for isolation and culture of MSCs from various sources, which makes it difficult to compare even the results of pre-clinical studies (95).

For clinical trials, a defined, clinical-grade cell product is required. As of now, over 80% of the MSCs used in clinical studies are expanded in media containing fetal bovine serum (FBS) (42), a crude and undefined mixture of growth factors and various bovine proteins. Beyond the unknown influences of various FBS preparations on the therapeutic effect of MSCs, even the potential risk of a pathogen transmission (viruses, prions) makes cells cultured with FBS not optimal for a clinical therapy (96). Based on the MSCs source, many other products used during the isolation process – including enzymes and growth factors – also derive from animal origins. Ideally, a product suitable for administration to a critically ill patient should be produced under current good manufacturing practice (cGMP)-conditions using defined xenogenic free chemicals.

Furthermore, it is crucial to accurately monitor growth and aging of MSCs *in vitro*. It is known that MSCs age during *ex vivo* expansion and that this influences biological properties of the cells (97). Different methods to determine the age of MSCs have been utilized. Most investigators and companies producing MSCs determine the passage number, an easy but very inaccurate parameter influenced by many factors (98). Therefore, it is not possible to determine if insufficient clinical effects are caused by real therapy failure or just by the fact that senescent therapeutic cells have been administered. A better way than counting passages might be the implementation of cumulative population doubling measurements (99) and biochemical assays, such as telomere attrition or *β*-galactosidase activity (100).

Prolonged culture of MSCs may also lead to genetic instabilities (101, 102). The spontaneous malignant transformation of MSCs observed in long-term culture experiments (103) has been proven to be an *in vitro* contamination artifact (104). However, the risk of tumorigenicity in MSC-based therapies is still under discussion (99). A direct tumor formation seems unlikely, as MSCs do not engraft. Indeed, in rats receiving MSCs for BPD no tumor masses were seen 6 months after therapy with the cells (16). The risks of increased tumor formation by long-term immunosuppression (99) or the previously discussed stem cell-stimulating effects remain unclear. A first meta-analysis of clinical trials using MSCs showed no increased tumor risk in over 1000 patients after 3–60 months after treatment (105). But as with every drug, definitive data regarding these issues can only be acquired in large clinical trials.

While MSCs are immune-privileged and as such enable allogeneic cell therapy, autologous cell therapy has also been advocated for. Autologous therapy may be associated with lower ethical and technical boundaries than therapy with allogeneic cells. Conversely, the autologous approach is logistically more challenging as it requires the manipulation of a fetal tissue (cord blood, cord stroma …) *ex vivo*. Therefore, each product will need to be subjected to a rigorous sterility and quality testing, which takes time, financial, and human resources as opposed to a ready-to-use off-the-shelf allogeneic cell product. It is also not yet clear for which preterm infant an autologous cell product should be processed. Furthermore, the autologous approach may not always be possible (outborn) or potentially deleterious (severe chorioamnionitis). These considerations will mature over time as knowledge and manufacturing technologies advance, allowing us to rationally determine the best possible cell product.

#### The Quest for a “Potency Assay”

One fundamental problem hampering the widespread use of MSCs in clinical trials is the absence of valid assays to assess their quality or “therapeutic potential” prior to usage.

In applications were the anti-inflammatory effects of MSCs are predominant (like GvHD), tests assessing the immunosuppressive potential of the therapeutic cells may overcome this obstacle (100, 106). In brief, MSCs are co-cultured with mitogen-stimulated allogeneic lymphocytes. They suppress the induced proliferation of the inflammatory cells to various extends via paracrine effects following direct cell-cell interaction. A simple automated cell count assesses the “therapeutic potential” of the MSC-population in this setting. An even faster and easier method uses the interleukin-10 stimulated expression of a specific subtype of the HLA-receptor complex (HLA-G) on the surface of MSCs (107) to assess their immunosuppressive potential. By now, it has not been validated if cells with higher anti-inflammatory potential *in vitro* lead to better therapeutic effects *in vivo* (100).

The situation for multi-factorial diseases affecting the lung – such as BPD – is, however, more complicated. As outlined previously, the mechanisms behind the beneficial effects of MSCs in BPD are complex and involve cytokines, the direct or paracrine interaction with resident cell types and maybe the transfer of mitochondria or exosomes. Therefore, the generation of such a simple functional assay is far ahead. *In vitro* approaches might involve the ability of MSCs to support the generation of alveolospheres out of murine alveolar type II cells in 3D organoid culture systems (108). The assessment of strain resistance in alveolar epithelial cells co-cultured with MSCs *in vitro* might be another interesting approach. Nevertheless, all these approaches remain far from an easy, fast, cheap, and reliable potency assay.

In summary, MSC therapies are promising and clinical conditions, such as BPD, urge for efficient treatment strategies. However, MSC therapies also represent a disruptive technology and for now, not a single trial investigated MSC products in man that met all current regulatory or cGMP criteria (95, 109). A safe and high-qualitative cell product to use in trials is still missing. As outlined recently in a position paper by Wuchter et al. (100), standardization and rigorous quality control of the production process is the *conditio sine qua non* for successful clinical testings using MSCs. If the product does not fulfill these criteria, how should we interpret the clinical results? Every disruptive technology is imperfect at the beginning and needs to evolve with experience and time. But it is imperative to do due diligence and obtain the best possible cell product before testing it in our most vulnerable patients.

## No Vessels, No Lung Growth: Progenitor Cells from the Endothelial Lineage

Simplification of the pulmonary vasculature is a hallmark of BPD (110), and angiogenesis is crucial for normal postnatal alveolar development (111). Hyperoxia-induced lung injury can be attenuated by increasing the pulmonary supply of angiokines like VEGF in rodents (111, 112). Accordingly, if vascular growth factors and lung angiogenesis contribute to the integrity of the lung, then vascular progenitor cells are appealing candidate cells likely to be involved in the same mechanisms.

After their first description as circulating cells in the peripheral blood by Asahara et al. (113), endothelial progenitor cells have been shown to promote the repair of damaged blood vessels in various disease models [reviewed by Mund and colleagues (114)]. They are further investigated as biomarkers of cardiovascular diseases [reviewed by Sen et al. (115)]. EPCs harbor the potential to form tube-like structures on matrigel matrices *in vitro*, home to ischemic sites *in vivo*, and augment angiogenesis by paracrine effects (116).

However, the population termed EPCs is not homogeneous, and the exact origin and definition of these cells remain unclear. A direct relationship of EPC subsets to the myeloid progenitor line has been described (117). Two groups provided evidence for a hierarchy within circulating EPCs and identified a specific subset named blood outgrowth endothelial cells (BOEC) (118) or endothelial colony forming cells (ECFCs) (119). This population, further referred to as ECFCs, is thought to contain the therapeutically active progenitor cells of the endothelial lineage (117). In contrast to the global EPC population, ECFCs lack expression of CD133 and CD115, exert high-clonal proliferative potential and harbor the ability to form vessels *de novo* when transplanted into immunodeficient SCID-mice [recently reviewed by Basile and Yoder (116)].

### Endothelial progenitors in BPD

Using a mouse model of BPD, Balasubramaniam et al. described that hyperoxia-induced lung damage depletes circulating EPCs and bone marrow-derived angiogenic cells (BMDACs) (120). Administration of BMDACs from healthy mice rescues the alveolar and vascular structure after O_2_ injury (31).

The role of circulating endothelial progenitors in the pathogenesis of BPD was further confirmed in studies with human infants. Borghesi and colleagues described that high numbers of ECFCs in the cord blood of preterm babies are associated with a lower risk to develop BPD (121). Interestingly, the blood counts of non-ECFC endothelial progenitors fail to predict or correlate to any disease associated with preterm birth (122), further substantiating the role of circulating ECFCs. Baker et al. also reported the association between low-ECFC counts and the development of BPD. They further showed that a decreased ratio between circulating progenitor cells with pronounced *in vitro* angiogenic potential (CPC) and those without (non-angiogenic, non-CPC) predicts the development of moderate or severe BPD (123). Moreover, ECFCs isolated from preterms are more prone to oxidative stress than cells from term infants (124). CdM from cord blood-derived ECFCs obtained from term infants promotes growth of the pulmonary vasculature, but fails to promote alveolar septation in bleomycin-induced lung injury (125).

The lung also harbors its own resident progenitor cells with vasculogenic capacity (30, 126, 127). Human fetal and neonatal rat lungs contain ECFCs with robust proliferative potential, secondary colony formation on replating, and *de novo* blood vessel formation. Exposure to hyperoxia *in vitro* and *in vivo* impedes ECFC function as exemplified by decreased proliferation, clonogenic, and angiogenic capacity. In experimental chronic hyperoxic lung injury in rats, administration of human cord blood-derived ECFCs restored resident lung ECFC colony- and capillary-like network-forming capabilities, lung function, alveolar and lung vascular growth, and attenuated pulmonary hypertension. At 10 months post-ECFC therapy improvement in lung structure, exercise capacity, and pulmonary hypertension persisted without signs of adverse effects (30). Comparable to MSCs, the benefit seems to be mediated by a paracrine effect since cell engraftment was minimal and CdM from ECFCs exerted similar therapeutic benefit to whole cell therapy.

### Room for a clinical application?

As of February 2015, no clinical trials using endothelial progenitor cells or their CdM as therapeutic agents in BPD are listed on www.clinicaltrials.gov. In the past, Wang et al. conducted two clinical trials in adult patients suffering from idiopathic pulmonary hypertension (NCT00641836 and NCT00257413). They used a heterogeneous preparation of autologous endothelial progenitors and demonstrated safety and feasibility as well as significantly increased exercise capacity and reduced pulmonary blood pressures 12 weeks after intravenous administration (128). A Canadian phase I study using EPCs transfected with endothelial nitric oxide synthase (eNOS) in seven patients has recently been completed (NCT00469027); final results are pending.

EPCs for therapeutic purposes could be isolated from easily accessible peripheral blood or cord blood without the ethical problems raised by a bone marrow puncture to obtain BMSCs. With the SCID-mouse transplantation assay, an excellent and reliable method assessing the functional capacity of ECFCs is available (117). However, the relatively complicated isolation and expansion process requires sophisticated (and expensive) media as well as many manual steps including the individual lifting of emerging colonies (117, 119).

As of today, no large-scale production technique has been developed to reliably isolate the quantities of cells required for clinical studies. Compared to MSCs, less is known about the behavior of EPCs or ECFCs *in vivo* and *in vitro*. Nevertheless, given the importance of angiogenesis for a large variety of diseases, cell-based vascular therapies will rapidly develop as our understanding of EPC biology advances in parallel with our knowledge in bioengineering and cell manufacturing.

## Not Stem Cells, Still Therapeutic: Amnion Epithelial Cells

Cells from the human amniotic epithelium (AECs) represent the third cell population that has been explored in experimental BPD. The amniotic membrane is widely used as an effective and low-immunogenic material to patch large skin defects (129). This tissue contains epithelial cells with distinct regenerative (130) and an anti-inflammatory potential (131) comparable to MSCs (132). AECs further possess the potential to differentiate along mesodermal, ectodermal, and endodermal lineages *in vitro* (130) and are considered “stem-like cells” (133).

In 2010, Moodley and colleagues described that i.v. injection of AECs abrogates lung fibrosis and inflammation in bleomycin-challenged immunodeficient mice. Furthermore, the cells homed and engrafted permanently into the damaged lung tissue, acquired the phenotype of alveolar type II cells, and started producing surfactant (133). In immunocompetent animals, similar effects without cell engraftment were observed (134). The potent anti-inflammatory and anti-fibrotic effects led to studies in fetal sheep with intraamniotic LPS-induced lung injury. Here, i.v. administration of AEC to the unborn lamb led to reduced lung inflammatory cytokines without significant improvements on lung structure (29).

In a study using *in utero* ventilation of fetal sheep to induce BPD-like changes in lung histology, Hodges et al. demonstrated significant improvements of the lung structure after combined i.v. and intratracheal administration of AEC during the ventilation procedure. Engraftment and transdifferentiation of AECs into alveolar type I and type II cells were noted. However, these rare events did not contribute to the overall impact of AECs in this animal model (28).

Currently, no clinical trials using AECs in pulmonary diseases are listed. However, AECs are investigated in a clinical trial for ocular limbal stem cell deficiency (135). Large quantities of the AECs can easily be produced from birth-associated tissues. But by now, no clear definitions and characterization regimen to define amniotic epithelial cells exist (136). Cells used in pre-clinical studies represent heterogenous populations, expressing a large variety of surface markers labeling pluripotent (SSEA-4), epithelial (cytokeratin-7, EpCam), and mesenchymal cells (CD73, CD90, CD166, among others) (29, 133). Nevertheless, first encouraging steps toward controlled, cGMP-conform isolation methods have been undertaken (137).

## Conclusion

Cell therapies represent the next paradigm shift in medicine. Unlike previous therapeutic game-changers, such as small molecules and biologics, cells are part drug and part device, which can sense diverse signals, interact with their environment, integrate inputs to make decisions, and execute complex response behaviors (138). These unique attributes of stem cells have been harnessed for organ regeneration. In the developing lung, various cell types including MSCs, EPCs, and AECs harbor the fascinating potential to provide pleiotropic therapeutic agents to protect from and restore lung damage. These cells are thus ideally suited not only for the treatment of a multi-factorial disease, such as BPD, but also for other complications of extreme prematurity.

Phase I trials with MSCs have already started and while the time is ripe for carefully designed early phase clinical trials, more progress is required to better understand the mechanisms of action and to optimize cell products. As with all disruptive technology, there is a steep learning curve in the beginning and the first product may be imperfect. Early on, common reference standards of the isolation and manufacturing process should be established to ensure uniformly high quality, effective and practical cell products. This will be crucial not only to ensure success of stem cell clinical trials but also to interpret and compare these trials. Finally, establishment of registries of all treated patients are imperative to ensure long-term follow-up.

Three excellent reviews further addressing the obstacles of bench-to-bedside transition of current stem cell therapeutics have been written by D. Prockop, S. Prockop, and I. Bertonello (95), G. Daley (139) and M. Fischbach, J. Bluestone, and W. Lim (138).

## Conflict of Interest Statement

The authors declare that the research was conducted in the absence of any commercial or financial relationships that could be construed as a potential conflict of interest.

## Abbreviations

AEC, amnion epithelial cell; ARDS, acute respiratory distress syndrome; ATP, adenosine triphosphate; BMDAC, bone marrow-derived angiogenic cell; BOEC, blood outgrowth endothelial cell; BOS, bronchiolitis obliterans syndrome; BPD, bronchopulmonary dysplasia; CD, cluster of differentiation; CDH, congenital diaphragmatic hernia; CdM, conditioned media; cGMP, current good manufacturing practice; COPD, chronic obstructive pulmonary disease; CPC, circulating progenitor cell; ECFC, endothelial colony forming cell; EPC, endothelial progenitor cell; EpCam, epithelial cell adhesion molecule; FBS, fetal bovine serum, FiO_2_ fraction of inspired oxygen; GvHD, graft-versus-host disease; IPF, idiopathic pulmonary fibrosis; MSC, mesenchymal stromal cell; PCR, polymerase chain reaction; PDGFR, platelet-derived growth factor receptor; SCID, severe combined immunodeficiency; SSEA, stage-specific embryonic antigen; TGF, transforming growth factor; VEGF, vascular endothelial growth factor.
